# Supplementary material for: Red Blood Cell Fatty Acid Profiles Are Significantly Altered in South Australian Mild Cognitive Impairment and Alzheimer’s Disease Cases Compared to Matched Controls
Source: Int J Mol Sci. 2023 Sep 15;24(18):14164. doi: 10.3390/ijms241814164 (PMC10531649; doi:10.3390/ijms241814164)
Supplement: Supplementary file 1 [file ijms-24-14164-s001.zip › ijms-2555701-supplementary.pdf]

Supplementary Tables

Supplementary Table S1. Pairwise comparison for both individual and combined gender for MCI and age and gender matched controls and AD and age and gender matched controls.

Fatty Acids	Gender	MCI Mean ± SEM	Con MCI Mean ± SEM	p value	AD Mean ± SEM	Con AD Mean ± SEM	p value
**14:0** Myristic acid	M+F	0.42 ± 0.02	0.37 ± 0.04	*0.41*	0.36 ± 0.03	0.28 ± 0.01	***0.03***
	M	0.39 ± 0.04	0.44 ± 0.10	*0.69*	0.40 ± 0.07	0.26 ± 0.02	*0.06*
	F	0.44 ± 0.03	0.34 ± 0.04	*0.08*	0.35 ± 0.04	0.29 ± 0.02	*0.19*
**15:0** Pentadecanoic acid	M+F	0.36 ± 0.03	0.35 ± 0.03	*0.92*	0.32 ± 0.03	0.32 ± 0.03	*0.99*
	M	0.36 ± 0.05	0.35 ± 0.05	*0.96*	0.33 ± 0.05	0.34 ± 0.06	*0.82*
	F	0.36 ± 0.04	0.35 ± 0.04	*0.93*	0.32 ± 0.03	0.31 ± 0.04	*0.97*
**16:0** Palmitic acid	M+F	25.48 ± 0.76	22.20 ± 0.77	***0.003***	23.36 ± 0.87	19.94 ± 0.35	***0.0004***
	M	25.40 ± 1.41	23.53 ± 1.41	*0.43*	26.00 ± 2.06	21.00 ± 0.84	***0.04***
	F	25.52 ± 0.94	21.48 ± 0.89	***0.001***	22.48 ± 0.85	19.58 ± 0.34	***0.006***
**18:0** Stearic acid	M+F	20.61 ± 0.52	19.09 ± 0.56	*0.06*	18.73 ± 0.47	16.91 ± 0.20	***0.001***
	M	20.54 ± 1.14	19.43 ± 0.95	*0.54*	19.76 ± 0.84	17.88 ± 0.37	***0.03***
	F	20.65 ± 0.56	18.90 ± 0.72	***0.04***	18.38 ± 0.55	16.59 ± 0.17	***0.008***
**20:0** Arachidic acid	M+F	0.81 ± 0.03	0.75 ± 0.03	*0.11*	0.74 ± 0.02	0.68 ± 0.02	***0.01***
	M	0.77 ± 0.04	0.71 ± 0.05	*0.40*	0.78 ± 0.06	0.73 ± 0.06	*0.58*
	F	0.84 ± 0.03	0.77 ± 0.03	*0.21*	0.73 ± 0.02	0.66 ± 0.01	***0.003***
**22:0** Behenic acid	M+F	3.14 ± 0.12	2.78 ± 0.11	*0.06*	2.66 ± 0.12	2.43 ± 0.06	*0.08*
	M	3.05 ± 0.26	2.80 ± 0.21	*0.56*	3.04 ± 0.30	2.61 ± 0.10	*0.31*
	F	3.18 ± 0.11	2.76 ± 0.12	*0.05*	2.54 ± 0.11	2.37 ± 0.06	*0.17*
**23:0** Tricosanoic acid	M+F	0.47 ± 0.02	0.41 ± 0.02	*0.11*	0.38 ± 0.01	0.38 ± 0.01	*0.69*
	M	0.44 ± 0.03	0.43 ± 0.06	*0.93*	0.42 ± 0.01	0.42 ± 0.01	*0.99*
	F	0.49 ± 0.02	0.40 ± 0.02	***0.02***	0.36 ± 0.01	0.36 ± 0.01	*0.59*
**24:0** Lignoceric acid	M+F	9.77 ± 0.26	8.99 ± 0.27	*0.08*	8.70 ± 0.23	8.42 ± 0.14	*0.34*
	M	9.52 ± 0.59	9.23 ± 0.48	*0.77*	9.42 ± 0.54	8.41 ± 0.25	*0.22*
	F	9.90 ± 0.26	8.86 ± 0.33	***0.03***	8.45 ± 0.23	8.43 ± 0.17	*0.92*
**Total SATFA**	M+F	61.05 ± 1.60	54.94 ± 1.67	***0.02***	55.26 ± 1 .64	49.35 ± 0.54	***0.001***
	M	60.46 ± 3.34	56.92 ± 3.17	*0.53*	60.14 ± 3.54	51.63 ± 1.18	***0.04***
	F	61.37 ± 1.78	53.87 ± 1.96	***0.004***	53.63 ± 1.71	48.59 ± 0.48	***0.01***
**16:1n-9** Palmitoleic acid	M+F	0.32 ± 0.02	0.31 ± 0.02	*0.87*	0.35 ± 0.07	0.29 ± 0.02	*0.47*
	M	0.30 ± 0.04	0.33 ± 0.05	*0.56*	0.53 ± 0.28	0.25 ± 0.02	*0.36*
	F	0.33 ± 0.03	0.30 ± 0.02	*0.56*	0.29 ± 0.02	0.31 ± 0.03	*0.63*
**18:1n-9** Oleic acid	M+F	16.08 ± 0.24	15.37 ± 0.27	*0.07*	16.06 ± 0.37	14.38 ± 0.16	***0.001***
	M	16.40 ± 0.47	15.05 ± 0.55	*0.10*	16.80 ± 1.12	14.80 ± 0.36	*0.16*
	F	15.90 ± 0.26	15.54 ± 0.30	*0.39*	15.82 ± 0.33	14.24 ± 0.18	***0.002***
**22:1n-9** Erucic acid	M+F	0.14 ± 0.01	0.15 ± 0.01	*0.07*	0.15 ± 0.01	0.17 ± 0.01	*0.15*
	M	0.14 ± 0.02	0.15 ± 0.01	*0.38*	0.13 ± 0.01	0.18 ± 0.01	*0.05*
	F	0.14 ± 0.01	0.15 ± 0.01	*0.08*	0.15 ± 0.01	0.16 ± 0.01	*0.56*

**24:1n-9** Nervonic acid	M+F	7.19 ± 0.20	7.06 ± 0.12	*0.56*	6.83 ± 0.21	6.67 ± 0.19	*0.54*
	M	7.05 ± 0.32	7.13 ± 0.22	*0.86*	7.01 ± 0.66	6.96 ± 0.39	*0.92*
	F	7.26 ± 0.24	7.03 ± 0.14	*0.31*	6.77 ± 0.19	6.57 ± 0.22	*0.52*
**Total MUFA**	M+F	23.72 ± 0.35	22.89 ± 0.33	*0.11*	23.39 ± 0.58	21.50 ± 0.29	***0.01***
	M	23.88 ± 0.72	22.66 ± 0.71	*0.31*	24.48 ± 1.89	22.19 ± 0.58	*0.30*
	F	23.63 ± 0.40	23.02 ± 0.36	*0.23*	23.02 ± 0.47	21.27 ± 0.33	***0.02***
**18:2n-6** Linoleic acid	M+F	4.76 ± 0.31	5.74 ± 0.30	*0.06*	5.41 ± 0.31	6.49 ± 0.22	***0.007***
	M	4.85 ± 0.64	5.94 ± 0.65	*0.37*	4.23 ± 0.55	6.22 ± 0.40	*0.05*
	F	4.71 ± 0.35	5.64 ± 0.32	*0.08*	5.80 ± 0.31	6.59 ± 0.27	*0.07*
**18:3n-6** γ-linolenic acid	M+F	0.07 ± 0.01	0.09 ± 0.01	***0.02***	0.08 ± 0.01	0.10 ± 0.00	*0.28*
	M	0.06 ± 0.01	0.07 ± 0.01	*0.49*	0.07 ± 0.01	0.10 ± 0.00	*0.09*
	F	0.07 ± 0.01	0.09 ± 0.01	***0.02***	0.09 ± 0.02	0.09 ± 0.01	*0.63*
**18:3n-3** α-linolenic acid	M+F	0.04 ± 0.01	0.07 ± 0.01	***0.005***	0.05 ± 0.01	0.09 ± 0 .01	***0.007***
	M	0.04 ± 0.02	0.07 ± 0.02	*0.31*	0.02 ± 0.01	0.06 ± 0.01	*0.05*
	F	0.04 ± 0.01	0.08 ± 0.01	***0.005***	0.06 ± 0.01	0.10 ± 0.01	***0.04***
**20:3n-6** Eicosatrienoic acid	M+F	0.75 ± 0.09	0.94 ± 0.08	*0.12*	0.93 ± 0.08	1.17 ± 0.07	***0.02***
	M	0.74 ± 0.18	0.85 ± 0.16	*0.73*	0.72 ± 0.16	1.07 ± 0.09	*0.06*
	F	0.75 ± 0.11	0.99 ± 0.09	***0.04***	0.99 ± 0.08	1.20 ± 0.09	*0.10*
**20:4n-6** Arachidonic acid	M+F	5.55 ± 0.77	8.61 ± 0.89	***0.01***	8.41 ± 0.94	11.75 ± 0.35	***0.001***
	M	5.19 ± 1.45	7.24 ± 1.66	*0.46*	6.03 ± 2.35	11.12 ± 0.61	*0.07*
	F	5.74 ± 0.93	9.35 ± 1.04	***0.004***	9.21 ± 0.94	11.97 ± 0.41	***0.01***
**20:5n-3** Eicosapentaenoic acid	M+F	0.49 ± 0.11	0.84 ± 0.12	*0.06*	0.96 ± 0.27	1.19 ± 0.18	*0.52*
	M	0.72 ± 0.29	0.87 ± 0.21	*0.73*	0.48 ± 0.21	0.79 ± 0.19	*0.22*
	F	0.37 ± 0.07	0.82 ± 0.15	***0.02***	1.12 ± 0.34	1.32 ± 0.23	*0.68*
**22:6n-3** Docosahexaenoic acid	M+F	2.48 ± 0.40	4.20 ± 0.49	***0.02***	3.76 ± 0.48	5.96 ± 0.35	***0.001***
	M	2.78 ± 0.90	3.74 ± 0.85	*0.53*	2.81 ± 1.17	4.55 ± 0.49	*0.08*
	F	2.31 ± 0.40	4.44 ± 0.61	***0.006***	4.07 ± 0.51	6.43 ± 0.36	***0.004***
**Total PUFA**	M+F	15.17 ± 1.79	22.05 ± 1.88	***0.02***	21.30 ± 2.02	29.02 ± 0.74	***0.001***
	M	15.62 ± 3.86	20.28 ± 3.70	*0.49*	15.38 ± 4.47	25.88 ± 1.48	***0.04***
	F	14.93 ± 1.95	23.01 ± 2.17	***0.004***	23.27 ± 2.09	30.07 ± 0.68	***0.01***
**Total n-3 PUFA**	M+F	4.05 ± 0.70	6.67 ± 0.74	***0.02***	6.47 ± 0.94	9.51 ± 0.58	***0.009***
	M	4.77 ± 1.65	6.18 ± 1.34	*0.59*	4.32 ± 1.80	7.37 ± 0.91	*0.06*
	F	3.66 ± 0.65	6.94 ± 0.92	***0.006***	7.19 ± 1.07	10.22 ± 0.62	***0.04***
**Total n-6 PUFA**	M+F	11.12 ± 1.15	15.38 ± 1.18	***0.02***	14.83 ± 1.22	19.51 ± 0.49	***0.001***
	M	10.85 ± 2.23	14.09 ± 2.42	*0.44*	11.06 ± 2.70	18.51 ± 0.73	***0.04***
	F	11.27 ± 1.36	16.07 ± 1.30	***0.006***	16.08 ± 1.24	19.85 ± 0.59	***0.01***
**n-3/n-6**	M+F	0.32 ± 0.03	0.41 ± 0.02	***0.02***	0.40 ± 0.04	0.49 ± 0.03	*0.11*
	M	0.35 ± 0.07	0.42 ± 0.04	*0.45*	0.33 ± 0.07	0.40 ± 0.04	*0.35*
	F	0.30 ± 0.03	0.41 ± 0.03	***0.02***	0.43 ± 0.05	0.53 ± 0.04	*0.18*
**PUFA/SATFA**	M+F	0.27 ± 0.04	0.43 ± 0.06	***0.01***	0.41 ± 0.05	0.59 ± 0.02	***0.001***
	M	0.29 ± 0.08	0.39 ± 0.09	*0.51*	0.28 ± 0.10	0.50 ± 0.04	***0.03***
	F	0.26 ± 0.04	0.45 ± 0.05	***0.004***	0.46 ± 0.05	0.62 ± 0.02	***0.01***

Supplementary Table S2. ROC curve sensitivity and specificity and likelihood ratios for controls and MCI and controls and AD cohorts

	**Cut off Values †**	**Sensitivity (%)**	**95% CI**	**Specificity (%)**	**95% CI**	**Likelihood ratio**	**Area Under Curve**	**p value ANOVA**
**Control and MCI**								
Palmitic Acid (C16:0)	> 21.36	90.0	68.30-98.77	70.0	53.7 - 83.44	3.0	0.85	**p < 0.0001**
Stearic Acid (C18:0)	> 18.31	85.0	62.11-96.79	72.5	56.11- 85.40	3.0	0.80	**p < 0.0001**
Arachidic Acid (C20:0)	> 0.73	75.0	50.90- 91.34	65.0	48.32 - 79.37	2.1	0.73	**p = 0.003**
Behenic Acid (C22:0)	> 2.64	90.0	68.30 - 98.77	62.5	45.80 - 77.27	2.4	0.80	**p = 0.0001**
Tricosanoic Acid (C23:0)	> 0.38	80.0	56.34- 94.27	50.0	33.80- 66.20	1.6	0.77	**p = 0.0006**
Lignoceric Acid (C24:0)	> 9.0	80.0	56.34 - 94.27	70.0	53.47 - 83.44	2.6	0.76	**p = 0.0009**
Oleic Acid (C18:1n9)*	> 15.0	80.0	56.34 - 94.27	65.0	48.32 - 79.37	2.2	0.79	**p = 0.0002**
Linoleic Acid (C18:2n6)	< 5.8	80.0	56.34 - 94.27	62.5	45.80 - 77.27	2.2	0.75	**p = 0.001**
γ-Linolenic Acid (C18:3n6)	< 0.07	95.0	75.13 - 99.87	57.5	40.89 - 72.96	2.2	0.81	**p < 0.0001**
Cis -8,11,14-Eicosatrienoic acid (C20:3n6)	< 1.11	80.0	56.34 - 94.27	40.0	24.86 - 56.67%	1.2	0.70	**p = 0.01**
Arachidonic acid (C20:4n6)	< 9.1	80.0	56.34 - 94.27	75.0	58.8 - 87.3	3.2	0.84	**p < 0.0001**
Linolenic acid (C18:3n3)	< 0.06	70.0	45.72 - 88.1	67.5	50.8 - 81.4	2.1	0.81	**p < 0.0001**
Docosahexaenoic acid (C22:6n3)	< 4.2	80.0	56.34 - 94.27	67.5	50.8 81.43	2.4	0.83	**p < 0.0001**
**Control and AD**								
Palmitic Acid (C16:0)	> 19.70	85.0	62.11 - 96.79	50.0	33.8 - 66.2	1.7	0.75	**p < 0.0001**

Oleic Acid (C18:1n9)*	> 14.78	85.0	62.11 96.79	62.5	45.8 77.27	2.3	0.79	**p = 0.0002**
Linolenic acid (C18:3n3)	< 0.06	85.0	45.72 - 88.11	67.5	50.87 81.43	2.1	0.71	**p = 0.009**
Docosahexaenoic acid (C22:6n3)	< 5.4	85.0	56.3 - 94.2	47.5	31.51 - 63.87	1.5	0.67	**p = 0.02**

p value represents that the AUC does actually discriminate between control, MCI and AD individuals for the fatty acid in question * Mixture of cis and trans isomers (Oleic and Elaidic acids) † Cut off points were arbitrarily chosen to maximise specificity and sensitivity values. The positive likelihood ratio represents the ratio between the probability of a positive test result given the presence of the disease (true positive rate) and the probability of a positive test result given the absence of the disease (false positive rate). Negative likelihood ratio represents the ratio between the probability of a negative test result given the presence of the disease (false negative rate) and the probability of a negative test result given the absence of the disease (true negative rate).
